# Neurocysticercosis and Hippocampal Atrophy: MRI Findings and the Evolution of Viable or Calcified Cysts in Patients With Neurocysticercosis

**DOI:** 10.3389/fneur.2019.00449

**Published:** 2019-04-30

**Authors:** Job Monteiro C. Jama-António, Clarissa L. Yasuda, Fernando Cendes

**Affiliations:** Department of Neurology, University of Campinas, UNICAMP, Campinas, Brazil

**Keywords:** neurocysticercosis, hippocampal atrophy, perilesional edema, magnetic resonance imaging, seizures, epilepsy, brain calcifications

## Abstract

Neurocysticercosis (NC) is the most common parasitic infection of the central nervous system (CNS). Several studies have reported an association between NC and mesial temporal lobe epilepsy (MTLE). We intended to evaluate the frequency of hippocampal atrophy (HA), clinical evolution and imaging findings in patients with calcified neurocysticercotic lesions (CNLs).

**Methods:** One hundred and eighty-one subjects (70 cases and 111 controls) were evaluated for the presence or absence of HA. We assessed the imaging findings, and the evolution of patients with NC treated or not with anthelmintics for NC.

**Results:** Hippocampal volumes were different between cases and controls (*p* < 0.001). Seventy percent of the cases presented HA. 52.2% of the patients without a history of anthelmintic treatment for NC had reports of epileptic seizures. There was an association between non-treatment and the later occurrence of epileptic seizures (*p* = 0.006). There was an association between perilesional edema on MRI and the presence of uncontrolled epileptic seizures (*p* = 0.004).

**Conclusions:** Hippocampal atrophy is frequent in patients with NCC. There was an association between no anthelmintic treatment in the acute phase of NC, perilesional edema, more pronounced hippocampal atrophy, and the occurrence of refractory seizures.

## Introduction

Neurocysticercosis (NC) is the most common parasitic infection of the central nervous system (CNS), caused by the larval form of Taenia solium (1). A frequent cause of symptomatic seizures and epilepsy worldwide (2). It is a severe public health problem in several regions of Asia, Africa, and Latin America (3–5).

The earliest documented descriptions of parasitic infection date back from Egyptian Medicine (6). Aristotle was the first to report the presence of cysticerci in animals, between 389 and 375 b.C (7). In ancient Greece, the disease was known as a pig disease (8). From the nineteenth century, it was clear that the disease was transmitted by man and not by animals as it was thought (7). NC was considered a public health problem after the second half of the twentieth century (8, 9).

Taenia solium is an enteroparasite belonging to the Platyhelminthes phylum, the Cestoda class, the Taeniidae family, the genus Taenia, and the solium species (7).

In its adult form, Taenia solium measures typically 2–4 meters in length and consists of scolex (head), neck (neck) and strobile (body) (8). Adults live on average 3 years and can live up to 25 years, housed in the digestive tract of humans (1).

The scolex invaginates and attaches to the mucosa of the small intestine. After cell division, they become adult tapeworm, which later eliminates gravid proglottid containing thousands of eggs, and thus, the cycle restarts (5). The man can act as an intermediate host, in this case, the human contamination with Taenia solium eggs is processed by (6, 7): External autoinfection; hetero-infection by ingestion of water or food, contaminated with T. solium eggs, disposed of in the environment by carriers; internal autoinfection may occur by intestinal antiperistaltic movements, making possible the presence of gravid proglottid or eggs in the stomach (1).

The oncosphere, when it reaches its final location, undergoes a vesiculation process and loses its aculeus, the invaginated scolex of the future adult, cysticercus Cellulosae (5), forms internally in the vesicle wall. Once established, the larval cysts, through mechanisms of immune evasion (complement inhibition, cytokine release, and masking of host immunoglobulins) actively avoid host immune response (8).

The Taeniasis-cysticercosis complex is a neglected tropical disease (NTD), usually associated with low socioeconomic development (6, 10). It is estimated that 50.000.000 individuals are infected each year (5, 11, 12).

Praziquantel and albendazole have been considered efficient in NC etiologic therapy (13). Therapy with albendazole or praziquantel is indicated in symptomatic individuals with viable cysts on CT or MRI and with positive evidence of immunological evidence for cysticercosis in CSF (2). The purpose of anthelmintic therapy is to try to reduce the duration of the neuroimmunological phenomena involved in NC (5). In most patients, it accelerates the degeneration of cysts and improves symptoms (14).

The clinical manifestations of NC are pleomorphic according to the viability of the parasite, occurring during or after the inflammatory process caused by the presence of dead or degenerated or calcified forms in the cerebral parenchyma (1, 8, 10, 15, 16). Epileptic seizures occur in up to 70–90% of the symptomatic cases of NC and generally represent the primary or unique manifestation of the parenchymatous form of the disease (17, 18). Patients with seizures invariably have prominent inflammatory infiltrate around the cysts, including the presence of pro-inflammatory cytokines and an altered blood-brain barrier (19).

The association between acute symptomatic epileptic seizures and NC is already well established, but the association between drug-resistant epilepsy and NC is still controversial (20). The majority of patients with acute symptomatic seizures in the active phase of the disease experience symptom remission in the next 3 to 6 months, together with the disappearance of the active lesions (20). However, degenerate as well as calcified cysts can lead to chronic epileptic seizures due to hippocampal sclerosis, probably triggered by an inflammatory process, recurrent epileptic seizures and local damage (2).

Neuroimaging and histology studies provide evidence that some nodules are not completely solid, but contain remnants of parasitic membranes that undergo periodic morphological changes related to remodeling mechanisms, thus exposing the host's immune system to the trapped antigenic material, causing recurrent epileptic seizures (2, 21, 22).

The presence of punctiform cerebral calcifications in the correct clinical scenario is mainly indicative of chronic cerebral NC (13). Often these calcifications are the only evidence of the disease (21). However, it is difficult to determine the causality of the relationship between epilepsy and NC, since calcifications are observed in asymptomatic individuals living in endemic areas (23). The use of CT and MRI produce objective evidence regarding the diagnosis of NC (24). These neuroimaging techniques have improved the accuracy of the diagnosis.

The first reports on NC findings on CT were published in 1977; since then, a number of studies have described in detail the different forms of the disease (25). The radiological descriptions allowed the development of clinical classifications of NC based on the topography and evolutionary stage of the lesions and were of great importance for the determination of the rational therapeutic approach in the different forms of the disease (25).

The imaging changes suggestive of NC are dependent on the development stage of the larva. Thus, in the CT the main ones are the following (25):

- Active phase (viable cyst): a cystic lesion is present, hypodense, with well-defined contours and with scolex inside (eccentric hyperdense nodule), without surrounding edema, nor contrast enhancement;
- Colloidal phase (cyst in degeneration): Presence of hypodense lesion, poorly defined, with surrounding edema, and enhancement in a ring or homogenous reinforcement in enhancement phase;
- Granular phase (onset of calcium deposition): small hyperdense nodules are present, surrounded by mild post-contrast enhancement edema;
- Calcification stage: The cysts appear as small hyperdense nodules, without perilesional edema or surrounded by post-contrast enhancement.

The mean interval between cysticercus death and radiologically perceptible calcification is approximately 25 months (26).

Cysticercus in intraventricular topography is not always detected by CT since its density is similar to CSF. Therefore, they can only be inferred by the distortion of the ventricular cavity (25).

In MRI the cysts appear with signal properties similar to CSF in both the T1 and T2 sequences. The scolex is usually visualized within the cyst as a high-density nodule, a “hole-with-dot” pathognomonic image, characterizing the viable phase (24).

Degenerating cysts (colloidal phase) present poorly defined contours due to edema (25). Some show ring enhancement after contrast administration. The cyst wall becomes thick and hypointense, with marked perilesional edema, best visualized in T2-weighted images (25). In the granular phase, cysts are visualized as ovoid signal areas in the T1 and T2 sequences, with surrounding edema or gliosis with hyperintense borders around the lesion (27). In the calcification phase, the cysts are usually not visualized (25). The susceptibility weighted imaging (SWI) sequence helps to visualize some calcifications. In the T1 and T2 sequences, calcifications can be visualized as small oval, hypointense images (10).

It has recently been demonstrated that calcified cysts can present perilesional edema and post-contrast enhancement, associated with recurrence of symptoms (28, 29).

These characteristics may serve as treatment-defining markers in these patients (30).

Hippocampal sclerosis is the most common structural brain injury associated with refractory mesial temporal lobe epilepsy (MTLE) (8, 31–37).

The histopathological mark of hippocampal sclerosis (HS) is the segmental loss of pyramidal (neuronal) cells, which may affect any segment of the “Ammon's horn,” mainly CA1 and CA4, associated with a severe pattern of astrogliosis in the hippocampal formation, including the dentate gyrus (34, 37).

In MRI, HS is characterized by reduced volume and loss of the internal structure of the hippocampus, better visualized in T1-weighted images, observed as hypointense signal, as well as an increased signal in T2-weighted images and FLAIR (37). On the other hand, quantitative volumetric studies allow an objective evaluation of the unilateral or bilateral atrophy of hippocampi, which makes them useful for research applications (24).

The co-existence of NC and TLE associated with HS is common in regions where NC is endemic (31, 33). It is believed that, as in febrile seizures, NC functions as an initial precipitating lesion that would later lead to hippocampal sclerosis (9, 17, 26, 38, 39).

In the last decades, several studies have suggested an association between NC and hippocampal atrophy (HA) (5, 40). New MRI techniques allowed more detailed evaluation of cystic lesions, inflammatory response, and other associated abnormalities (14).

Our objective was to evaluate the frequency of hippocampal atrophy (HA) in patients with NC calcified lesions (NCC), describe the symptomatic evolution of patients treated and not treated for NC, and identify parenchymal alterations associated with the occurrence of epileptic seizures.

## Methods

### Ethics Statement

All participants signed the informed consent form before performing the magnetic resonance (MRI) examination. This study was approved by the ethics and research committee (CEP-UNICAMP); CAAE Number: 55942116.5.0000.5404.

### Clinical Data

We included 181 subjects (70 cases and 111 controls). Individuals aged 18 years and older, followed by our outpatient's epilepsy clinic or headache clinic at the State University of Campinas (HC-UNICAMP) clinic hospital. We defined our primary variable of interest as the presence of active or calcified cysts in Computed Tomography (CT). We extracted information on the presence of active or calcified cysts from reports of radiological examinations that were available in the medical records. When they were not available, we assigned a qualified neurologist to evaluate CT scans, taking into account Carpio's criteria (41). Patients with a history of follow-up due to neurotuberculosis, neurotoxoplasmosis, tuberous sclerosis, and surgery for temporal lobe epilepsy were excluded from the study. We also excluded patients whose diagnosis was not confirmed after CT evaluation. Seventy patients participated in the study; 48 had no history of treatment for NC, 22 had a history of active cysticercosis and received treatment for NC between the years 1993–2013. The localization of cysts (calcified) observed on CT, were defined as temporal and extratemporal. Patients with multiple calcifications were classified as temporal lobe if they had a temporal lobe lesions, regardless of the location of the other lesions. The extratemporal category was assigned if the location of the lesion was only outside the temporal lobe. Regardless of whether or not they were treated for NC and the number of antiepileptic drugs used, those who had at least one seizure during the evaluation year were considered as individuals with uncontrolled seizures, and those who were 1 year or more without seizures were considered as with seizure control.

All participants performed MRI for volumetric analysis of hippocampus. Those who did not have recent MRI exams (< 2 years before the study) were invited to perform further MRIs.

### Protocol of MR Image and Visual Analysis

Patient and control MRI scans were performed on a 3-T Philips Intera Achieva scanner (Philips, Best, The Netherlands), with acquisitions in the coronal, sagittal and axial planes, with coronal sections obtained perpendicularly along the axis of the hippocampal formation, to better study this structure.

### MRI Acquisition Protocol

✓Coronal images: (a) T2-weighted images multi-echo (3 mm thickness, repetition time (TR) = 3,300 ms, echo time (TE) = 30/60/90/120/150 ms, matrix = 200X180, field of view (FOV) = 180X180); (b) T1-weighted images “inversion recovery” (3 mm thickness, TR = 3,550 ms, TE = 15 ms, inversion time = 400, matrix = 240X229, FOV = 180 × 180), (c) Fluid Acquisition Inversion Recovery (FLAIR) Suppression of fat, 4 mm thickness, TR = 12,000 ms, TE = 140 ms, matrix = 180 × 440, FOV = 200 × 200);
✓Axial images: FLAIR images (Fat suppression, 4 mm thickness, TR = 12,000 ms, TE = 140 ms, matrix = 224 × 160, FOV = 200 × 200);
✓T1 weighted volumetric images: 1 mm isotropic voxels, acquired in the sagittal plane (1 mm thick, flip angle = 8°, TR = 7.0 ms, TE = 3.2 ms, matrix = 240 × 240, FOV = 240 × 240);
✓T2-weighted volumetric images: isotropic voxels of 1.5 mm, acquired in the sagittal plane (TR = 1,800 ms, TE = 340 ms, matrix = 140X140, FOV = 230 × 230);
✓SWI (susceptibility weighted imaging) and gadolinium T1 weighted images for patients with a history of active cysticercosis.

### Volumetry of the Hippocampus

Patients and controls were matched for age and sex (with similar distribution about age, *p* = 0.211 and gender, *p* = 0.693). A group of 111 healthy subjects was used as controls (55.9% female, age 18–80 years, mean 45.05).

We selected the 3D T1-weighted images for volumetry. These were compressed in the *neuroimaging informatics technology initiative* (NIFTI) format through a web interface. Subsequently, the hippocampal volumes were obtained automatically using the volBrain online program (http://volbrain.upv.es). The automatic analyses were performed without knowledge of clinical data. All individual hippocampal values were corrected for total intracranial volumes. All values obtained were transformed into Z-score. The Z-score values of the corrected volumes or asymmetry index (defined by the ratio of the smallest to the largest hippocampus), which were equal to or lower than −2 were considered indicative of HA (Table 1).

**Table 1:** Distribution of the Z-score values and asymmetry index of the hippocampus volumes of patients who had HA.

**Number**	**Side of the atrophy**	**Right Z-score**	**Left Z-score**	**Index of asymetry (Z-score)**
1	L	−1.35	−2.97	0.79 (−9.94)
2	L	−1.26	−2.09	0.88 (−5.53)
3	R	−1.6	1.23	0.77 (−11.04)
4	L	−0.55	−2.45	0.78 (−10.37)
5	B	−3.44	−3.40	0.95 (−1.74)
6	L	−0.46	−1.09	0.91 (−3.99)
7	L	0.57	−0.25	0.90 (−4.29)
8	R	−2.03	−0.42	O.86 (−6.23)
9	R	−0.03	−0.58	0.92 (−3.34)
10	L	0.59	−2.98	0.65 (−16.71)
11	L	−1.62	−3.21	0.78 (−10.71)
12	R	−2.47	−0.43	0.82 (−8.34)
13	L	−1.55	−1.71	0.94 (−2.12)
14	R	−5.02	−0.39	0.57 (−20.95)
15	R	−0.69	0.47	0.91 (−3.83)
16	R	−3.66	2.97	0.52 (−23.26)
17	R	−3.4	−1.72	0.83 (−7.70)
18	L	−1.44	−4.18	0.66 (−16.16)
19	L	0.31	−3.01	0.67 (−16.01)
20	R	−3.43	−1.08	0.78 (−10.46)
21	B	−2.30	−2.04	0.99 (−0.08)
22	L	0.11	−2.55	0.72 (−13.25)
23	L	0.86	−3.39	0.71 (−14.03)
24	B	−5.54	−3.88	0.80 (−9.29)
25	L	−0.97	−2.21	0.84 (−7.49)
26	R	−3.33	−1.13	0.79 (−9.68)
27	R	−0.73	0.98	0.87 (−6.06)
28	L	−1.21	−1.92	0.89 (−4.89)
29	L	0.43	−0.11	0.92 (−3.15)
30	R	−3.40	0.22	0.68 (−15.20)
31	L	1.87	−1.32	0.72 (−13.27)
32	L	−0.32	−4.66	0.54 (−22.06)
33	L	−1.45	−3.02	0.79 (−9.81)
34	B	−2.59	−3.03	0.90 (−4.40)
35	B	−5.26	−2.03	0.65 (−16.67)
36	L	−1.10	−1.37	0.94 (−2.51)
37	L	−0.59	−1.25	0.90 (−4.16)
38	R	1.81	0.19	0.85 (−6.90)
39	R	−3.09	0.83	0.67 (−15.69)
40	L	−0.38	−3.50	0.66 (−16.71)
41	B	−3.25	−3.17	0.96 (−1.40)
42	R	−2.00	0.58	0.78 (−10.10)
43	L	1.30	−0.29	0.84 (−7.21)
45	L	−0.49	−1.31	0.89 (−4.93)
46	B	−2.10	−4.68	0.65 (−16.63)
47	L	−0.89	−1.43	0.91 (−3.77)
48	R	−0.44	0.36	0.94 (−2.25)
49	R	0.26	1.11	0.94 (−2.31)

*L, left; R, right; B, bilateral; HA, hippocampal atrophy*.

### Visual Analysis of Images

In patients with a history of NC treatment, a visual analysis of the MRI examinations acquired on a 3T (as described previously) or in a 2.0T (Elscint Prestige, Haifa, Israel) scanner was performed by two investigators (JMCJA and FC). In addition, 54 MRIs were analyzed with the objective of evaluating the evolution of the cysts through the images. Th MRI acquisitions of these 54 patients were carried out between the years 2004 to 2018. The findings were correlated with the occurrence of a seizure described in the medical record during the period of MRI (equal to or < 1 month). Further details are in Tables 2, 3.

**Table 2:** Distribution of study variables and the level of significance.

	**Overall (*****n*** **=** **181)**						
	**Patients (*****n*****:70)**				**Controls (*****n*** **=** **111)**		***P-*value**
Mean age ±*SD*	47.14 (± 12.98)				45.05 (± 12)		0.211
**Gender**							
Male n (%)	33 (47.1)				49 (44.1)		0.693
Female n (%)	37 (52.9)				62 (55.9)		
	**Treated for NC (*****n*** **=** **22)**		**Untreated for NC (*****n*** **=** **48)**		**Controls (*****n*** **=** **111)**		
Family history (%)	4 (18.18)		13 (27.0)		–		0.060
**Hippocampus mean volume/SD**	Right	Left	Right	Left	Right	Left	**0.001**
	3.69 cm^3^ 0.57	3.43 cm^3^ 0.59	3.44 cm^3^ 0.56	3.38 cm^3^ 0.58	3.92 cm^3^ 0.34	3.84 cm^3^ 0.31	
Seizure-recurrence (%)	8 (36.3)		36 (75.0)		–	–	**0.003**
**Calcification**							
Temporal left n (%)	6 (27.27)		8 (16.66)				
Temporal right n (%)	2 (9.09)		7 (14.58)		–	–	
Temporal bil. n (%)	8 (36.36)		3 (6.25)				
Extratemporal n (%)	6 (27.27)		30 (66.25)				
Hip. Atrophy n (%)	15 (68.18)		34 (70.83)		–	–	0.825

*The Kruskall-Wallis test showed a significant difference between the groups (p = 0.001). There was an association between non-treatment for NC and recurrence of seizures (p = 0.003, chi-square test). Hip, Hippocampal; bil, Bilateral; SD, Standard Deviation. Significant p-values are in bold*.

**Table 3:** Main findings of visual MRI analysis of patients treated for NC and report of seizures in the same period.

**Number**	**Year of initial symptoms/year of MRI**	**Perilesional gliosis**	**Perilesional edema**	**Contrast enhancement**	**Hippocampal atrophy**	**Diffuse cerebral atrophy**	**Ventricular dilatation**	**Seizure occurrence**
1	1994/2015	Yes	No	No	Yes	No	No	No
2	1994/2013	Yes	No	No	Yes	No	No	No
3	2010/2015	Yes	No	Yes	No	No	No	No
4	2012/2017	Yes	No	No	No	No	No	No
5	1999/2016	Yes	Yes	Yes	Yes	No	No	Yes
6	2013/2017	Yes	Yes	Yes	No	No	No	Yes
7	1998/2015	Yes	Yes	Yes	No	No	No	Yes
8	1994/2011	Yes	Yes	Yes	No	No	No	Yes
9	1998/2017	Yes	Yes	Yes	No	No	No	Yes
10	2010/2010	Yes	Yes	Yes	No	No	No	Yes
11	1995/2016	Yes	Yes	No	No	No	No	Yes
12	2007/2017	Yes	Yes	Yes	Yes	No	No	Yes
13	1993/2011	No	Yes	No	Yes	Yes	No	Yes
14	1993/2015	Yes	No	Yes	No	No	No	No
15	2013/2016	Yes	Yes	Yes	Yes	Yes	Yes	Yes
16	2009/2011	Yes	Yes	Yes	Yes	Yes	Yes	Yes
17	2002/2011	No	No	No	No	No	No	Yes
18	1993/2013	Yes	Yes	No	No	No	No	No
19	1993/2011	Yes	Yes	No	No	No	No	Yes
20	2009/2011	Yes	Yes	Yes	Yes	No	No	Yes
21	2004/2012	Yes	Yes	Yes	No	No	No	Yes
22	2006/2012	Yes	Yes	Yes	No	No	No	No

*In this table, we illustrate the gender, the date of the first MRI, the month and the year of the exam with parenchymal alteration, and the record of epileptic seizures in the same period*.

### Statistical Analysis

Data analysis was performed using SPSS software version 23 for mac. First, we did an exploratory analyses, measuring the frequency of categorical data and descriptive statistics for quantitative data.

To compare the groups (controls and cases), we performed a normality test (Kolmogorov-Smirnov). Then, the Mann-Whitney or Kruskall-Wallis test was performed to analyze numerical variables. Multivariate analysis was performed on numerical variables (controls, treated, and not treated for NC). The chi-square or Fisher's test were used to analyze the categorical variables. The significance was determined as *p* < 0.05 for all analyses.

## Results

**Figure 1:**
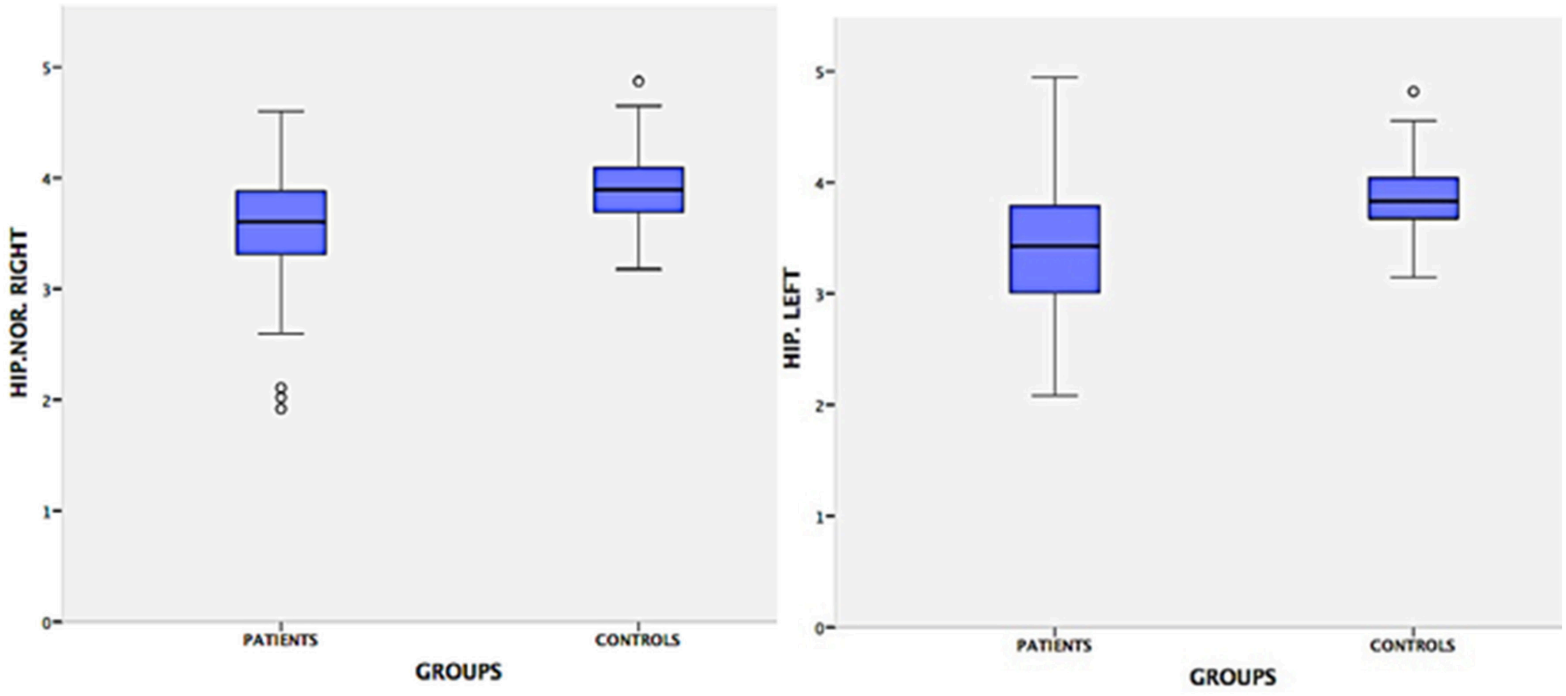
Hippocampal volumes of patients and controls. This graph demonstrates that there is a difference in the size of the hippocampus of NC patients compared to healthy controls. The Mann-Whitney test showed a significative difference between the hippocampal volume of patients and controls (*p* = 0.001). Evidence of a possible relationship between NC and hippocampal atrophy. HIP.NOR.RIGHT: hippocampus normalized right; HIP.LEFT: hippocampus left. **Patient–**Hip. Right, Mean = 3.50 cm; *SD* = 0.57; Range = 2.68; Hip. Left, Mean = 3.36 cm; SD = 0.60; Range = 2.88; **Controls–**Hip. Right, Mean = 3.92 cm, *SD* = 0.34, Range = 1.92; Hip. Left, Mean = 3.84 cm, *SD* = 0.31; Range = 1.89.

**Figure 2:**
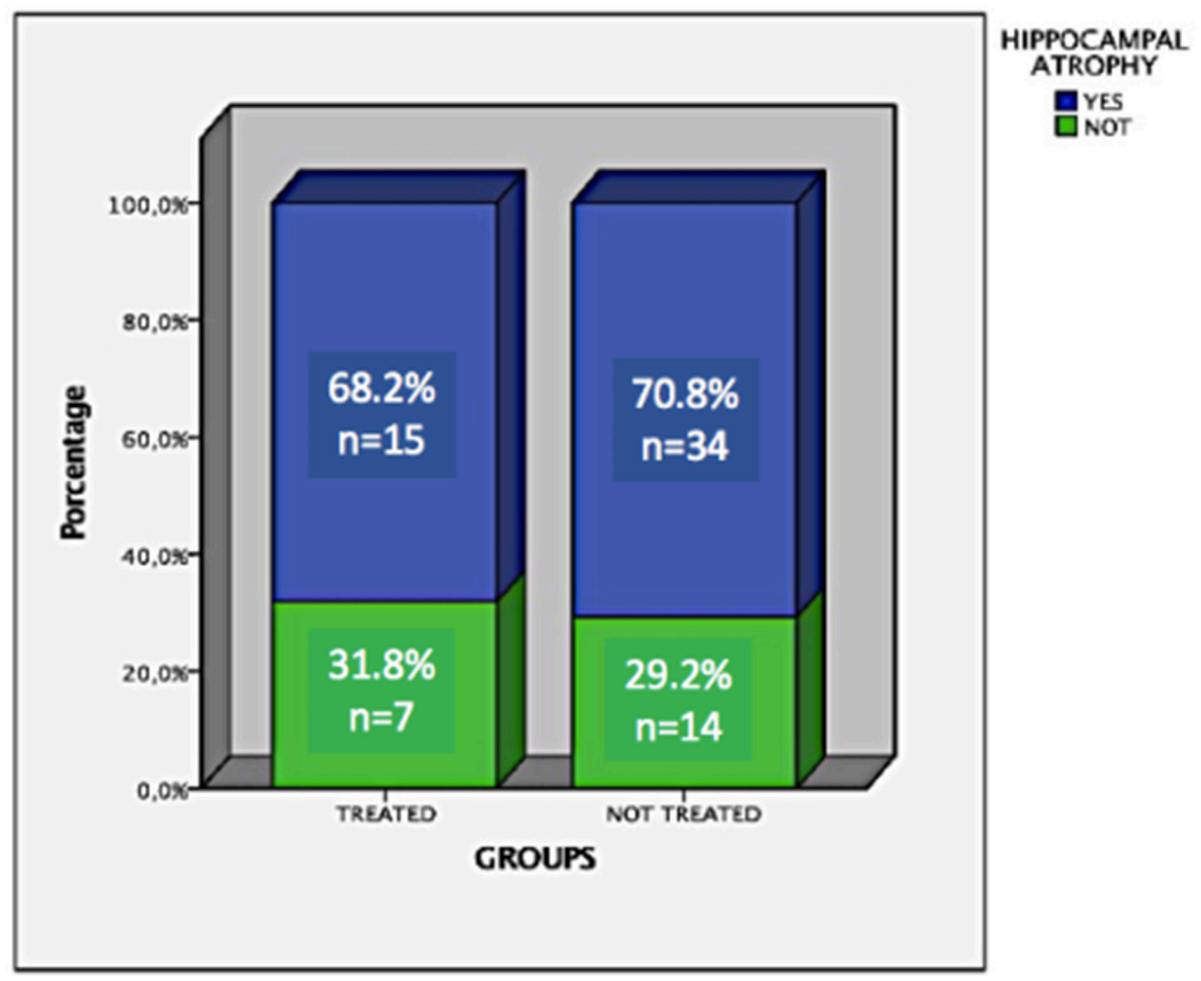
Frequency and percentage of hippocampal atrophy in patients treated and untreated for NC.

**Figure 3:**
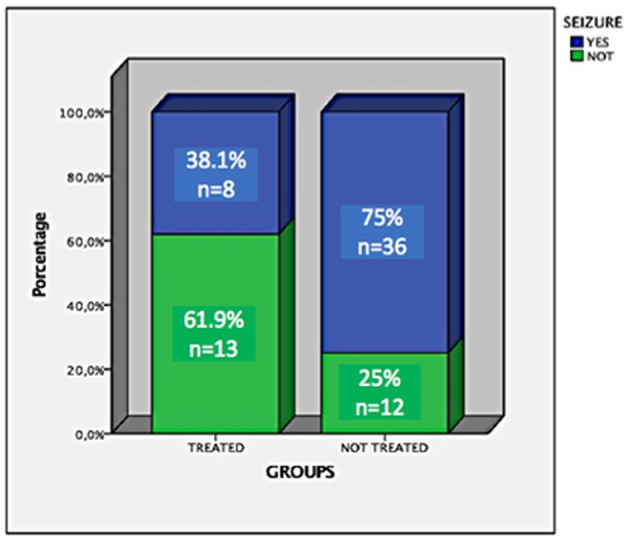
Frequency and percentage of uncontrolled seizures in patients treated and untreated for NC.

From an original sample of 211 participants, we included 181 (111 controls and 70 cases). Ninety-nine were female, mean age = 45.8, ±12.4. Hippocampal volumes of the controls were significantly different from the cases by the Man-Whitney test (*p* < 0.001, Figure 1). In a subgroup analysis (controls, patients treated, and patients untreated for NC), we observed that there was only a difference of controls compared to patients untreated for NC (*p* = 0.001; Figures 2, 3). Groups had a similar gender distribution (*p* = 0.693).

### Case Analysis

**Figure 4:**
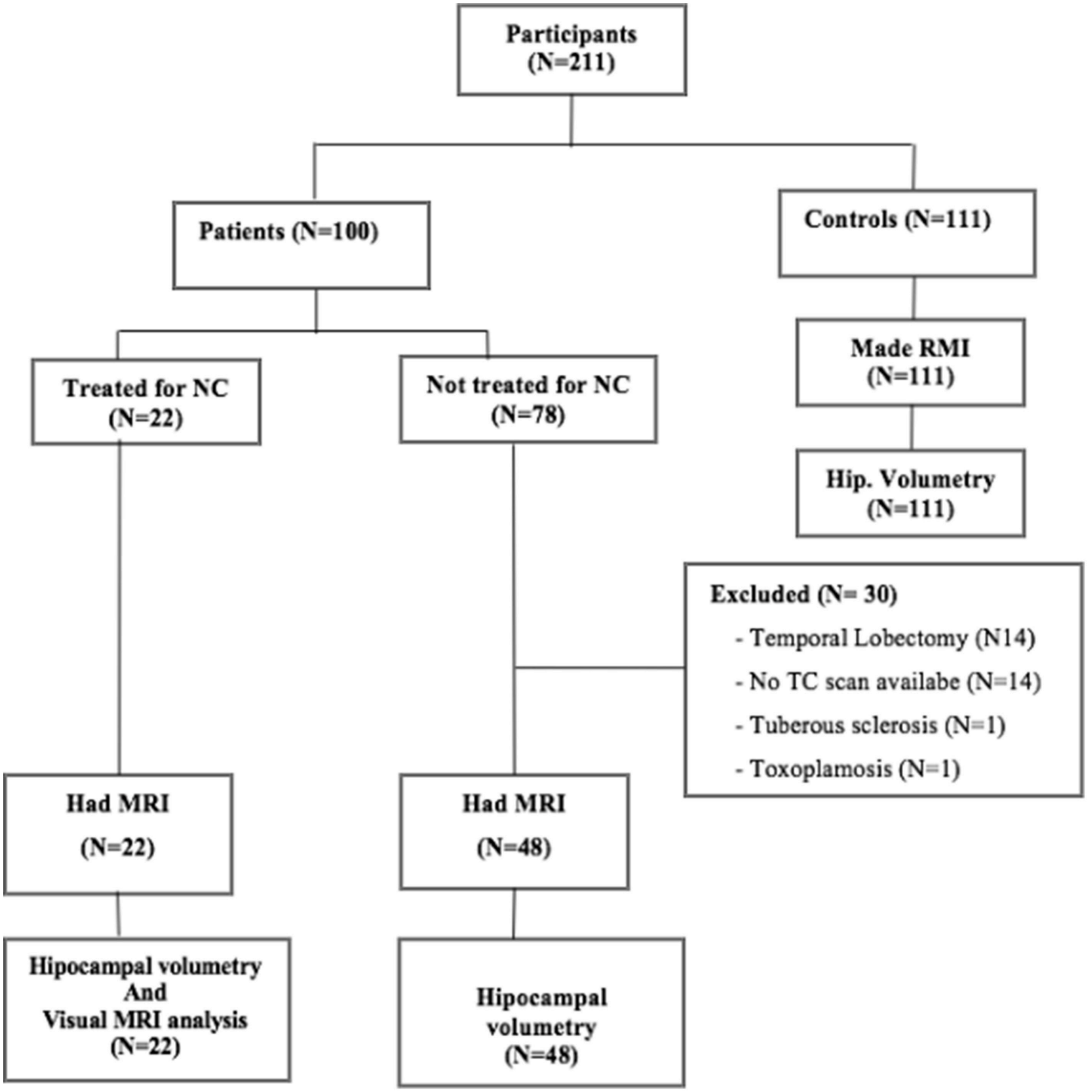
Study flowchart.

Of the 70 cases, 22 (31.4%) were treated for NC, 48 (68.6%) were not (Figure 4). There was no difference in the volume of the hippocampi of treated and untreated patients for NC (*p* = 0.225). There was no age difference (*p* = 0.220) or sex distribution (*p* = 0.401) between groups.

### Location of Calcifications

In 34/70 (48.6%) the NC calcifications were localized in the temporal lobe: 14/34 (20%) in the left temporal lobe, 9/34 (12.9%) in the right temporal lobe and 11/34 (15.7%) in both temporal lobes. In 36/70 (51.4%) the NC calcifications were localized in extratemporal regions.

### Number of Calcifications

Twenty-six of 70 (37.1%) patients had one to two parenchymal calcifications, 24/70 (34.29%) had three to five calcifications, 14/70 (20%) had six to twenty calcifications, 6/70 (8.57%) had more than twenty calcifications.

### Clinical Manifestation

Only 1/70 (1.4%) of the patients did not present seizures in the acute phase or in the follow up.

### Hippocampus Atrophy

Forty-nine of the 70 (70%) patients presented HA. There was no difference between HA and the localization of calcifications (*p* = 0.2, Fisher exact test). Fifteen of the 22 (68.18%) patients treated and 34/48 (70.83%) of the untreated patients had HA. There was no association between the frequency of HA and treatment for NC (*p* = 0.83); however, patients who did not receive anthelmintic treatment in the acute phase had significantly smaller hippocampal volumes (*p* = 0.0001). There was no association between HA and sex (*p* = 0.96). Only 17/70 had a family history of epilepsy (*p* = 0.06). Further details are in Table 2.

### Epileptic Seizures Report

forty-four of the 69 (68.8%) patients had uncontrolled epileptic seizures; 36 of these 44 (81.8%) did not receive anthelmintic treatment for NC in the acute phase of the disease. There was an association between the uncontrolled epileptic seizures and non-treatment for NC (*p* = 0.003).

Thirty-four of the 44 (77.3%) patients with uncontrolled seizures presented HA and remaining 22.7% had well controlled seizures (*p* = 0.065).

### MRI Visual Analysis

Here we analyzed the patients with more than one MRI exam, and whose presence of viable cysts was confirmed by imaging tests.

Fifty-four MRI exams of 22 patients performed between 2004 and 2018 were analyzed. The average duration of follow-up was 15 years (range of 4–23 years). Five of 22 (22.72%) patients had active cysts in at least one of the exams. Two of 22 (9.09%) had ventricular dilatation, and 3/22 (13.63%) had diffuse cerebral atrophy.

Nineteen of 22 (86.4%) patients presented perilesional gliosis in at least one of the calcified lesions. However, there was no association between the presence of gliosis and the occurrence of seizure (*p* = 0.963). Sixteen of 22 (72.7%) presented perilesional edema around at least one of the calcified lesions. There was an association between the presence of perilesional edema and the occurrence of seizure in the weeks before the MRI exam (*p* = 0.004). Fourteen of 22 (63.6%) had contrast enhancement around at least one of the calcified lesions. There was no association between contrast enhancement and the occurrence of seizures (*p* = 0.51). Eight of these 22 (36.4%) had hippocampal atrophy. Further details are in Table 4.

**Table 4:** Distribution of the main findings of visual MRI analysis and the level of significance in relation to the seizure occurrence.

**Variables. n (%)**	**Patients with uncontrolled seizures**	**Patients with seizure control**	***P*-value**
**Patients (*****n*** **=** **22)**			
Perilesional gliosis	13 (68.42)	6 (31.57)	0.963
**Perilesional edema**	14 (87.5)	2 (12.5)	**0.004**
Contrast enhancement	10 (71.42)	4 (28.57)	0.510
Hippocampal atrophy with other signs of HS.	6 (75.00)	2 (25.00)	0.490
Diffuse cerebral atrophy	3 (100)	–	–
Ventricular dilatation	2 (100)	–	–

*There was an association between perilesional edema and recurrence of seizures (p = 0.004; Fisher's test). Significant p-values are in bold*.

### Evolution of Patients With Active Cysts

We evaluated an average of 3 exams for each patient, performed between 3 to 11 years after the first examination of the acute phase of cysticercosis (viable or degenerating cysts). Five of these 22 presented active cysts in initial MRIs.

**Figure 5:**
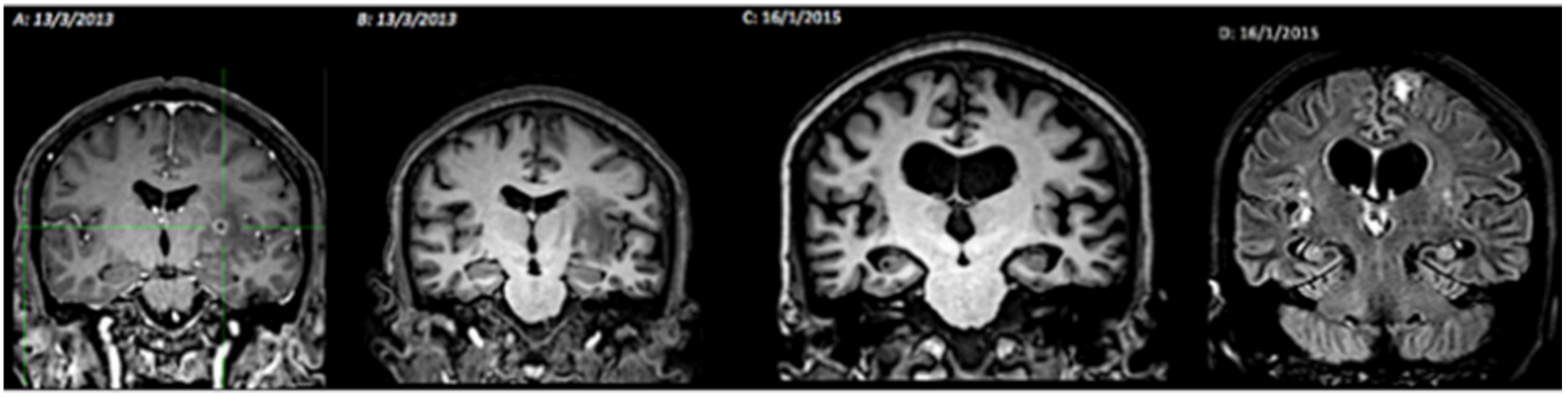
Illustration of the relationship between neurocysticercosis and hippocampal atrophy. MRI evolution (2013–2015). **(A)** T1-weighted coronal image, post contrast, with cysts in the colloidal phase, contrast enhancement, no atrophy of the hippocampus; **(B)** T1-weighted coronal image, with perilesional edema, without hippocampal atrophy; **(C)** T1-weighted coronal image, showing diffuse cerebral atrophy, including bilateral hippocampal atrophy; **(D)** FLAIR sequence, with hyperintense signal in the hippocampus (atrophy), and left frontal and perinsular hyperintense lesions.

In one case, we observed the occurrence of hippocampal atrophy 2 years after the beginning of the cyst degeneration process that was not present before (Figure 5).

**Figure 6:**
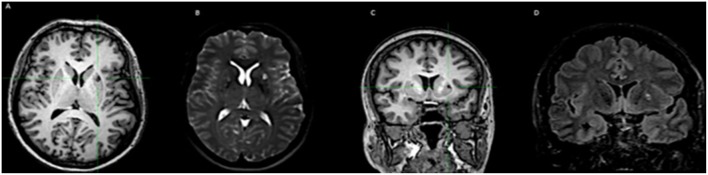
Illustration of the calcified NC associated with perilesional edema. **(A–C)** T1-weighted images, with calcification in the putamen. **(B–D)** Images in T2 and FLAIR, with edema around the calcification.

**Figure 7:**
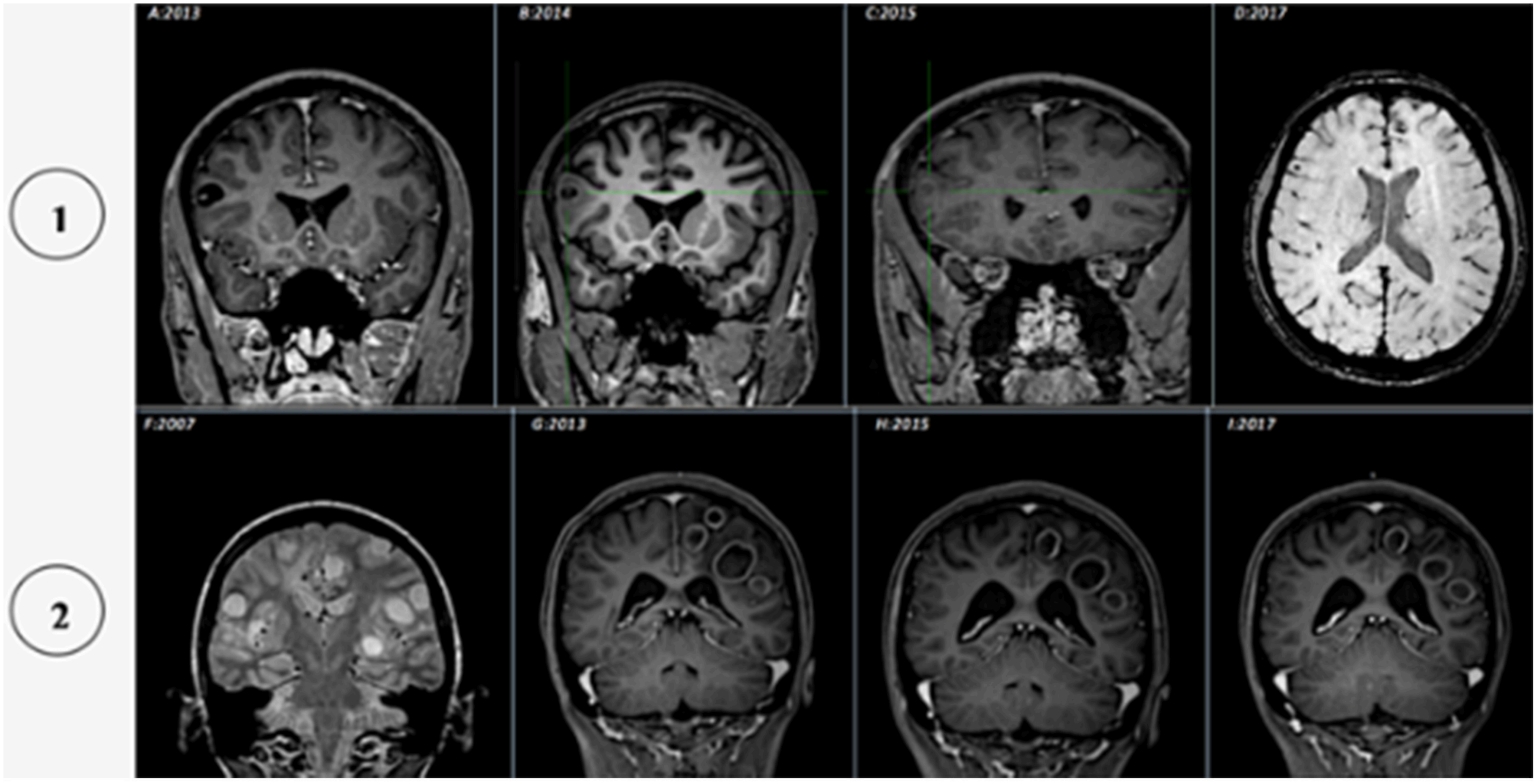
Illustration of the cysticercus evolution in two patients, from vesicular to granular or calcified: (1) (2013–2017) **(A)** T1-weighted coronal image, post contrast, showing cysts in vesicular phase (with scolex); **(B)** T1-weighted coronal image, without contrast, showing cysts in colloidal phase (perilesional edema); **(C)** T1-weighted coronal image, contrast enhancement, showing cysts in granular phase (mild contrast enhancement); **(D)** SWI MRI, calcification phase (hypointense image). (2) (2007–2017) **(F)** T2-weighted coronal image, showing cysts in vesicular phase (without scolex); **(G)** T1-weighted coronal image, post contrast, presenting cysts in colloidal phase (perilesional edema and contrast enhancement); **(H–I)** T1-weighted coronal image, post contrast, granular phase (mild contrast enhancement).

The evolution of the cysts was variable (Figures 5–7): The process of calcification occurred between 3 and 4 years after the diagnosis of active cysts. However, in one specific case, the degenerative cysts maintained enhancements for about 10 years later (2007–2017, details in Figure 7).

## Discussion

We observed a high frequency of hippocampal atrophy in patients with NC (70%), suggesting a possible association between NC and HA. This possibility has been considered for years by several authors, who have studied such an association (5, 38, 40, 42, 43).

In a study that sought to determine the relationship between HA, NC and seizure semiology in epileptic patients, the authors observed that HA is more frequent in patients with MTLE and calcified NC, compared to patients with extratemporal epilepsies (40). In another population study, the authors, when assessing the association between NC and HA in older adults living in an endemic area found a high prevalence of HA (68%) in patients with calcified NC compared to controls (26). In another study, the authors evaluated 324 patients with MTLE-HS undergoing temporal lobectomy, and they found a high prevalence of calcific NC, 126/324 (38.9%) (4). Another case-control study found a high frequency of calcified NC in patients with MTLE-HS (31).

During the last decades, anecdotal reports and small series of cases have brought this association to the attention of the medical community, describing patients with drug-resistant MTLE-HS whose neuroimaging studies showed granular or calcified cysticerci located in the hippocampus or neighboring tissues (2). In some cases, the pathological exams revealed HS with neuronal loss in the CA1 and CA4 layer, and gliosis, as well as the presence of an intense inflammatory reaction in the brain tissue around the calcified parasites (2).

In the active form of cysticercosis, inflammation involves the parasites, and is the most common mechanism for the occurrence of seizures in the acute phase on NC (3). This inflammation is due to the aggregation of mononuclear lymphocytes, plasma cells and variable numbers of eosinophils at the lesion site (3). Experimental studies have suggested that the injection of *Taenia* granuloma material into the mouse hippocampus is highly epileptogenic, supporting the involvement of the hippocampus by the inflammatory responses of the brain of the degenerating cysticerci (38).

Current evidence shows that the relationship between NC and MTLE-HS has always coexisted in endemic areas (38). However, the extent of this occurrence remains to be determined, so in many cases it is considered as “dual pathology” (2, 9, 38). Most of the information on this association comes from series of patients with MTLE-HS that suggest a cause-and-effect relationship (2, 4, 26). As in the febrile seizures during childhood, NC would act as an initial precipitating lesion, which would cause damage to the hippocampus, leading to loss of neurons and synaptic reorganization of the cellular elements (9, 14, 38, 40, 44). In this conjecture, it has been suggested that cysticerci can lead to HS because they cause repetitive inter-ictal discharges, recurrent clinical and subclinical seizures or possibly epileptic status, which results in MTLE-HS, and in turn aggravate seizures (9, 26, 38). These parasites do not necessarily have to be located within the limbic system (17), suggesting a deleterious remote effect of NC-induced reactive seizures in hippocampal neurons (38).

On the other hand, parasitic cerebral lesions may lead to inflammation-mediated hippocampal damage associated or not with genetic susceptibility (9, 42, 45). In this view, the periodic remodeling of cysticercus occurs with the exposure of parasitic antigens bound to the host's immune system, which does not require recurrent seizures as a causal factor (9, 26). Although this has not been demonstrated in humans, there is experimental evidence showing that repeated exposure to endotoxin and increased levels of pro-inflammatory cytokines correlate with hippocampal damage, supporting the hypothesis of inflammation-mediated atrophy or hippocampal damage (2, 26).

Another possibility is that the presence of HS in patients with NC may be only a coincidence (31, 42), which in our view is less likely, given the high prevalence reported in this and other studies (40).

In cysticercosis calcification, recurrent seizures may result from inflammation related to exposure of the host immune system to parasitic remains (2). In the vicinity of the lesion, the tissue reaction usually consists of astrocytic gliosis and a small border of demyelination. Neurons are affected variably and tend to undergo degenerative changes (3). It seems reasonable to assume that the inflammation at the stage of nodular calcification is similar to that of the colloidal stage. Acute and recurrent seizures, if repeated, may cause additional hippocampal damage. Also, degenerate and calcified cysticerci can directly induce hippocampal sclerosis by damage mediated by local or remote inflammation of hippocampal neurons causing refractory epilepsy (2).

The format of this study did not allow us to directly establish a cause and effect relationship between NC and HA, however, in a case of active NC, we were able to demonstrate that hippocampal atrophy was related to the degenerate cysticercus, due to an inflammatory reaction. There was no HS before degeneration of the cysticercus, however, 3 years later the MRI signs of HS were observed (Figure 2). In this case, the hippocampus has probably been directly affected by the inflammatory response and gliosis that develops around the cyst and/or adjacent areas (38).

In addition to the high frequency of MTLE-HS in our patients with calcified NC, there was an association between the absence of anti-helmintic treatment in the acute phase of NC and later uncontrolled epileptic seizures, as well as smaller hippocampal volumes, something that may infer that anthelmintic treatment works as a protective factor. MTLE-HS is often pharmacoresistant and many patients reach seizure-free status only after surgical treatment (9).

The mechanism of involution of cysticercosis, which, contrary to what was previously thought, the final step (degeneration and calcification), is not completely inert (21, 46). It is known that NC is a potential cause of refractory epilepsy and that the presence of perilesional gliosis contributes to epileptogenicity (30). About half of the patients with only calcified lesions and recent ongoing seizures, developed perilesional edema at the time of seizure recurrence (28). A plausible explanation for the occurrence of perilesional edema may be that they are not all alike and may differ in the amount, in the form of calcium deposition, in the degree of antigens recognized by the host, in the level of residual inflammation, or by the proximity of a blood vessel (46), which favors the occurrence of perilesional edema. On the other hand, genetic factors may also be related (20). Some attest that this is due to dysfunction of the blood-brain barrier, probably due to the presence of inflammation and/or perilesional gliosis conditioned to the host's response to the newly recognized or released parasite antigen and/or to the positive regulation of the immune response of the host (28). Histopathological examination of calcification associated with multiple episodes of perilesional edema revealed significant inflammation, which supports the concept that edema is inflammatory in nature (28).

Some authors argue that perilesional edema is the result of an inflammatory process directed at the sequestered parasite antigen (47), and therefore advocates specific measures to limit the inflammation process, which can be used to treat or prevent complications (28).

Another hypothesis is that perilesional edema occurs as a consequence of seizure activity (13). However, there are differences between edema associated with a flurry of seizures and perilesional edema, the first being more diffuse, with no defined maximum area of activity, presumably caused by the loss of fluid by damaged cells, while the second presents a peak, almost always accompanied by contrast enhancement, probably of vasogenic origin (28). In general, edema around calcification after seizures is considered an evident form of injury that is probably epileptogenic (20, 48). A previous study concluded that the presence of edema is a predictor of recurrence of seizures (30).

We conclude that there is a high frequency of AH in patients with NC, which may suggest an association between both. In addition, there was an association between no anthelmintic treatment and the later occurrence of uncontrolled seizures and smaller hippocampi, as well as between perilesional edema and seizures near the time of the MRI exam.

## Ethics Statement

This study was approved by the ethics and research committee (CEP-UNICAMP); CAAE Number: 55942116.5.0000.5404.

## Author Contributions

All authors listed have made a substantial, direct and intellectual contribution to the work, and approved it for publication.

### Conflict of Interest Statement

The authors declare that the research was conducted in the absence of any commercial or financial relationships that could be construed as a potential conflict of interest.
